# Developing Hierarchical Schemas and Building Schema Chains Through Practice Play Behavior

**DOI:** 10.3389/fnbot.2018.00033

**Published:** 2018-06-25

**Authors:** Suresh Kumar, Patricia Shaw, Alexandros Giagkos, Raphäel Braud, Mark Lee, Qiang Shen

**Affiliations:** ^1^Department of Computer Science, Aberystwyth University, Aberystwyth, United Kingdom; ^2^Department of Electrical Engineering, Sukkur IBA University, Sukkur, Pakistan

**Keywords:** Dev-PSchema, practice play, schemas, action sequencing, schema chains, play and playthings, modeling of behavior

## Abstract

Examining the different stages of learning through play in humans during early life has been a topic of interest for various scholars. Play evolves from practice to symbolic and then later to play with rules. During practice play, infants go through a process of developing knowledge while they interact with the surrounding objects, facilitating the creation of new knowledge about objects and object related behaviors. Such knowledge is used to form schemas in which the manifestation of sensorimotor experiences is captured. Through subsequent play, certain schemas are further combined to generate chains able to achieve behaviors that require multiple steps. The chains of schemas demonstrate the formation of higher level actions in a hierarchical structure. In this work we present a schema-based play generator for artificial agents, termed Dev-PSchema. With the help of experiments in a simulated environment and with the iCub robot, we demonstrate the ability of our system to create schemas of sensorimotor experiences from playful interaction with the environment. We show the creation of schema chains consisting of a sequence of actions that allow an agent to autonomously perform complex tasks. In addition to demonstrating the ability to learn through playful behavior, we demonstrate the capability of Dev-PSchema to simulate different infants with different preferences toward novel vs. familiar objects.

## 1. Introduction

Humans are capable of learning within different environments and of extending their knowledge to new situations. As new experiences are gained, our capacity to understand the world and to adapt to changes within it strengthens. We are also capable of generalizing experiences and of repeating successful behaviors that were previously expressed, in related situations. Developing this capability in robots is one of the major goals of roboticists. Modeling requires an in-depth understanding of how we learn from experiences and how we develop our knowledge.

We learn different behaviors throughout our entire life, beginning with initial sensorimotor experiences that develop to high level cognitive reasoning over time and through a series of stages. Piaget's cognitive theory (Piaget and Cook, 1952) proposes different learning stages in humans, supporting the idea of constructivism. He believed that children develop a variety of cognitive skills at different ages. The first stage of the cognitive developmental theory is referred to as the Sensorimotor stage, where learning is focused on the sensorimotor experiences of the infants. Experiences that are gained at this stage are related to the infants' own actions and the associated sensory outcomes.

At the early stage of their life infants spend much of their time playing, a behavior closely coupled with their ability to learn (Pramling Samuelsson and Johansson, 2006). They explore their own actions and understand the resulting effects. Owing to this strong correlation, play is seen as an important part of cognitive development (Nicolopoulou, 2010). In addition, play provides a foundation for academic and social learning (Hirsh-Pasek and Golinkoff, 2008).

Infants appear to be very interested in their surrounding environment and tend to perform a wide variety of free play activities in order to explore it. Their actions are not constrained by any predefined rules other than those related to physical capabilities. Nevertheless, physical constraints do help them to scaffold learning, as the infants gradually understand the different elements related to their behaviors. At the sensorimotor stage infants learn all relevant elements of the actions and sensory information that are associated with their experiences (Baillargeon, 1994). Apart from exploratory play, infants demonstrate exploitation behaviors during play. They explore the environment and the objects in it, extending their learning into novel and identical environments through a process of generalization (Baldwin et al., 1993; Welder and Graham, 2001).

In robotics, we aim to develop robots that are autonomous and capable of operating within dynamic environments and adapting to the changes that occur. The robotic agents ought to be able to re-use any previously acquired experiences in order to perform sufficiently in novel situations and under new circumstances. They should also be capable of learning from different experiences and through performing different tasks. Indeed, developmental robotics concentrates on modeling infant learning so that robots learn and adapt in similar ways to humans. More specifically, modeling the play behavior of infants provides a mechanism for robots to explore and discover new knowledge, acting as a driver for learning (Lee, 2011).

To develop a robot that learns from experiences and adapt, several learning systems have been proposed to make it learn from active and passive experiences (Drescher, 1991; Montesano et al., 2008; Krüger et al., 2011; Aguilar and y Pérez, 2015; Petit et al., 2016; Kansky et al., 2017). Drescher (1991) proposed a learning mechanism based on Piaget's schema mechanism. Following Drescher's proposed system, Sheldon (2013) introduced PSchema, a schema-based system that focuses on learning from sensorimotor associations, where learning outputs are formulated as schemas that contain sensory information that is received before and after an action is performed. At the beginning, the system learns a set of basic actions by considering only the proprioception of the hand. This process is referred to as bootstrapping and is inspired by the reflexive movements of infants toward distant stimuli (Piaget and Cook, 1952). The next action to be performed is selected by a mechanism responsible for the calculation of the excitation associated with each action, in an intrinsic motivation fashion. Being an open-ended learning system, PSchema is capable of creating action sequences in addition to generalizing experiences (Sheldon and Lee, 2011). However, they are only created when the targeted conditions are provided by the user.

Krüger et al. (2011) introduced a learning model for autonomous agents using sensorimotor experiences, allowing an agent to interact with the real world and to develop hierarchical knowledge. The latter is termed Object Action Complexes (OACs) and constitutes the means by which the system enables behavior planning. OACs are essentially tuples of (i) an action, (ii) the sensory-state transition (initial to final predictable state) caused by the action, and (iii) the reliability of predicting the resulting state in the environment. Different problem-related learning algorithms can be used to learn OACs. Wörgötter et al. (2009) presented the implementation of an OAC model related to their robotic application. In their work, OACs are learned through a supervised learning method and are tested on a robotic arm. The goal of the experiment they presented is to move an object from one point to another by removing obstacles along the path. Their results show that the OACs model is capable of planning and making predictions. However, goals for the planning are set up by the user rather than the agent itself. This limits the agents capability for performing open-ended learning and encouraging continuous play behavior. Moreover, the capability to generalize experiences is recognized as future work, limiting its performance within novel environments.

Also inspired by Piaget's theory, Aguilar and y Pérez (2015) developed a schema-based learning system called Developmental Engagement-Reflection (Dev-ER) for autonomous agents. Learning consists of schemas that contain preconditions, an action and the postconditions, which are results of applying the action on the preconditions. The model is employed by a virtual agent in a 3D virtual environment, where it can passively observe the environment by moving its head and by fixating to interesting objects. The latter are found by the use of an attention process based on an interest value of the perceived objects. Interest values depend upon three aspects; pre-programmed preferences, number of object features (properties) and virtual emotional interest in the object. The agent is initially provided with two reflexive saccade schemas, through which it develops its knowledge by interacting in the environment to create more schemas. The attention system helps the agent to demonstrate the playful behavior. However, the model is not capable of planning in order to achieve a goal within the environment, or to exploit a sequence of actions in order to achieve a state in the environment which is not possible with a single action.

Most recently, Kansky et al. (2017) developed a schema-based deep learning network, based on the generative model of a Markov Decision Process (MDP). The objects are represented by lists of fixed binary properties, where an object may or may not have a given property in the environment. The network can perform planning toward maximizing the reward from the initial state as it matches the goal state in the environment. To evaluate the network, an experiment is performed using the environment of the classic arcade video game Breakout. In the game, a ball is used to gradually break a brick wall positioned at the top of the screen by being repeatedly bounced between the bricks and the player's paddle that moves horizontally on the bottom of the screen. Points are awarded every time a brick is hit by the ball, which is enough to break it from the wall, without missing the ball. The performance of the schema network is compared with two other deep learning network models; Asynchronous Advantage Actor-Critic (A3C) and Progressive Networks (PNs), in different experiments containing different variations in the environment. The results show that the proposed network outperforms the others in all the variations of the environment, capable of generalizing and adapting what it has learnt to variations of the environment. However, the network still needs a large amount of training to achieve a better result.

The above learning models are comparable to the schema-based mechanism proposed in this work. However, some of these systems do not offer open-ended play behavior (Krüger et al., 2011; Kansky et al., 2017) and some do not offer planning behaviors to achieve a desired or given state in the environment (Sheldon, 2013; Aguilar and y Pérez, 2015). Here, we present an intrinsically motivated open-ended learning and play generator system, termed Dev-PSchema. By employing it, an agent plays and learns that a ball can be grasped and moved to a different location and disappears when dropped in a hole. The system can learn from a small number of experiences and can combine them in order to construct higher level reusable chains of actions to represent more complex hierarchical behaviors. An excitation mechanism triggers learning by exploratory play during which the system generalizes schemas and re-uses them in novel situations. Moreover, with a change in the excitation parameters, different individual infants are simulated, a feature that is absent to all of the above discussed learning models. Finally, the system is sufficiently abstract and can be used with different platforms without making any major design changes.

In Dev-PSchema, each schema consists of the pre and post states of the environment (i.e., the world) related to a high-level action. The term high-level defines the actions without underlying motor/joint movements.

The work presented in this paper draws inspiration from Piaget's schema mechanism. An initial implementation of this mechanism is given in Drescher (1991), with a model based on the sensorimotor stage, i.e., the first learning stage from the four stages of cognitive development outlined by Piaget. Learning at this stage is believed to be associated with motor actions that are performed by the developing infant. Based on this idea, the schema system simulates an agent which learns from its sensory experiences that result from motor actions, and uses the knowledge that was previously acquired to interact with the environment. The mechanism has no concept of persistence of objects while associating the sensory cues, i.e., touch, sound and vision, with the performed actions in order to generate new behaviors.

In Section 2 we present Dev-PSchema and the experiments along with the results. In Section 3 we discuss the system's capability to express different behaviors due to variations in the excitation parameters and to learn high-level actions by developing schemas chains. Finally, in Section 4 we provide a conclusion about our findings in the light of developmental psychology.

## 2. Dev-PSchema and experiments

Dev-PSchema builds on PSchema, a previously developed system by Sheldon (2013), and simulates an agent within an environment capable of interacting with it. By considering simulated sensory information as well as actions that the agent can perform, the system is capable of learning action-effect correlations. These are represented as schemas and constitute the knowledge the agent gains by interacting with objects within the environment. At the beginning, the system starts with a basic set of action schemas, referred to as bootstrap schemas (details are found in Kumar et al., 2016a,b), stating the actions that can be performed without describing the preconditions associated with them. Subsequently, the system is free to start applying the schemas in the environment and, by interacting with objects, to learn new ones while expressing playful behaviors. As such, the system is considered a play generator that allows infant behaviors and learns to emerge through playing.

As the agent interacts with the environment, new schemas are added to record new experiences or unexpected outcomes from actions, incorporating the preconditions from which the effect was experienced. These new schemas contain a set of sensory information, the behavior and its predictions in the environment. We refer to the sensory information as preconditions, the behavior as action and the sensory predictions or results as postconditions. Thus, a schema is a tuple that consists of an action and the sensory information from both before and after the execution of the action, as preconditions and postconditions respectively. Any unpredicted effect of actions, as described by the schema used by the agent at any time, leads to the generation of new experiences that are also captured as new schemas. For instance, this happens when the postconditions of a schema do not match the resulting phenomena of the schema's action. Note that Dev-PSchema operates in discrete time; the system records observations before and after the execution of an action. Counting actions that are performed from the beginning of an experiment indicates the time-steps. During a single time-step, the system records all available observations to form the preconditions, executes an action and finally records observations again to form the postconditions. A chain of schemas is also executed within a single time-step.

Table 1 shows an example of a schema that was learnt after grasping an object using an initial bootstrap schema. Here the sensory information and the actions are defined as high-level abstractions, rather than the sets of raw sensor data and motor commands that they reflect. When in use, the system is connected to a body^1^ via a low-level system that is responsible for the generation and availability of perceptions and actions for the schemas. In the case of real robotic hardware, the low-level system translates the schema actions into appropriate motor activities allowing the agent to interact with the environment. Although schemas could be used to represent low level actions and sensory information the focus here is on high level playful behavior.

**Table 1:** An example of the concrete “Grasp” schema.

**Grasp Schema**		
**Preconditions**	**Action**	**Postconditions**
Color “Green” at x = 2, y = 2		Color “Green” at x = 2, y = 2
Shape “Sphere” at x = 2, y = 2	Grasp	Shape “Sphere” at x = 2, y = 2
Touching object		Holding object

In order to generate play behaviors within an environment, attention and novelty are important (Mather, 2013). Dev-PSchema employs an excitation mechanism that provides action selection by identifying those object-action pairs that are most interesting to the agent considering their postconditions. Selection of interesting object-action pairs depends upon the agent's preferences. Whereas such preferences are affected by novelty and habituation (i.e., familiarity) of the environment. The system provides exploratory play behaviors to interact with the objects and learn outcomes related to different actions performed on them. Note that the objects in the system are defined with the visual perceptions containing underlying properties. On one hand, the system is capable of exploring an object by performing actions associated with it. On the other, the system has the ability to switch between objects as necessary, ensuring the evaluation of the transferability of any learned knowledge while encouraging further explorations.

Furthermore, the system is able to create sequences of schemas in order to achieve a distance state (i.e., set of postconditions) that may not be feasible with a single schema (chains are discussed later in Section 2.2). The agent will create new schemas and chains of schemas from existing schemas wherever possible following the execution of a schema or chain. The process of creating new schemas following interaction resembles the adoption process where a subject learns new knowledge building upon an existing knowledge base as described by Piaget and Cook (1952).

Below we describe the key components that allow the generation of schemas and schema chains and therefore the development of the learning. In particular the excitation calculator (Section 2.1) and the chaining mechanism (Section 2.2).

### 2.1. Excitation calculator

Considering all objects in the environment, as they are perceived via sensory information, the agent calculates the excitation of each available schema in order to find the most interesting one to be executed with respect to the current perceived environment referred as world state. Calculating the excitation is based on the similarity, novelty and habituation assigned to each schema, the total excitation of a schema is a weighted combination of these three factors. Varying the weights allows the generation of different play behaviors (Oudeyer et al., 2007; Ugur et al., 2007), that could correspond to different simulated infants or to behaviors expressed within varying external environmental conditions (e.g., playing in a familiar or unfamiliar setup).

In particular, similarity is designed to favor schemas related to previous interactions with a given object, whereas novelty increases the excitation value for new objects or objects that have not been interacted. Subsequently, habituation decreases the interest the agent has for an object that is frequently used for interactions over time. Obviously, novelty and habituation are in contradiction by which the agent switches its attention from objects that have been explored to those that propound novel interactions. Note that although the terminology used in this work is based on that of developmental psychology, the meaning is not an exact match. Therefore, a precise definition of all three of such factors of excitation are given below.

#### 2.1.1. Similarity

This factor is used to describe the degree of resemblance between the object-specific perceptions that are captured at the end of an action and those that constitute the postconditions in each of the previously learned schemas. It is calculated by matching individual properties of an object, such as color or shape.

Such that

(1) $$\begin{array}{l} Similarity=\frac{\sum_{i=1}^{C(\rho )}\underset{1\leq j\leq C(\zeta )}{\max}[Sim(\rho_{i},\;\zeta_{j})]}{C(\rho )} \end{array}$$

where

$$\begin{array}{l} Sim(\rho_{i},\zeta_{j})=\{\begin{array}{ll} 1, & \rho_{i}≅\zeta_{j}\\ 0.5, & \rho_{i}\sim \zeta_{j}\\ 0, & \rho_{i}≁\zeta_{j} \end{array} \end{array}$$

returning the similarity between the *i*^*th*^ property of the object's perception ρ, that is ρ_*i*_, and the *jth* property of the schema's object perception (ζ_*j*_). *C*(ρ) is the count of the number of properties in the perceived object and *C*(ζ) is that of in a schema object perception. If a property appears in both states but the values are different, then *Sim* will return a partial match, i.e., 0.5^2^. The result, in short, is the ratio between the sum of all maximum similarities calculated by *Sim* and the total number of properties in the perceived object.

The result is a number between 0 and 1, with 1 indicating an exact match. Although each property is compared with all properties found in all schemas, only the one with the maximum similarity measure is considered.

#### 2.1.2. Novelty

This is calculated by considering how frequently perceptions that describe an object are confirmed as postcondition in schemas, in connection to the running time-step:

(2) $$\begin{array}{l} Novelty=(1+cos(4.75*\tau_{1}))/2 \end{array}$$

where

(3) $$\begin{array}{l} \tau_{1}=\frac{C(O_{s})}{C(O_{e})} \end{array}$$

with *C*(*O*_*s*_) being the number of times the object perception *O* appeared in schemas and *C*(*O*_*e*_) being that *O* was captured in the environment.

**Figure 1:**
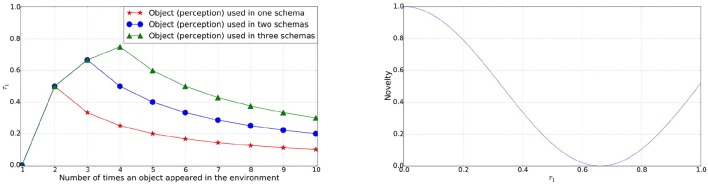
**Left**: Value of τ_1_ for an object perception used in 1, 2, or 3 schemas continuously against the number of times it appeared in the environment. **Right**: Novelty of an object perception over the range of value for τ_1_.

The novelty factor is designed to express a smooth curve for values between 0 and 1 for τ_1_, as shown in Figure 1. The cosine is scaled between 0 and 1, with the period reduced such that at τ_1_ = 1.0 the value is 50%. Novelty of the perceived object transitions from the maximum to the minimum and then back up to the 50% over the values of τ_1_ from 0 → 1. Initially the novelty of the newly perceived object will be the maximum. As the object is played with more frequently or appears more in schemas its novelty reduces. If the object is not played with for a longer period of time, its novelty again increases.

#### 2.1.3. Habituation

**Figure 2:**
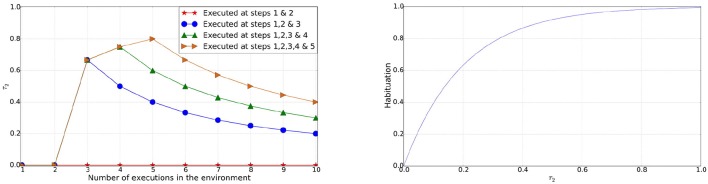
**Left**: Value of τ_2_ for an object perception in schemas used in execution steps [1 & 2], [1, 2 & 3], [1, 2, 3 & 4], and [1, 2, 3, 4 & 5] against the execution steps. **Right**: Habituation of an object (perception) over the range of values for τ_2_.

This factor depends on how recently schemas containing the object perception are used in the environment. The agent is expected to be more habituated, hence less interested, with a situation that reoccurs after interacting with the environment. This is inspired by developmental psychology, where infants become habituated with objects or events after a period of exploration or observation (Sigman, 1976; Hunter et al., 1983; Kirkham et al., 2002; Colombo et al., 2004). Habituation at a given time-step is given by

(4) $$\begin{array}{l} \tau_{2}=\{\begin{array}{l} \frac{1}{n}\sum_{i=1}^{n}\frac{T_{s_{i}}}{T_{c}},\;if\;n>0\\ 0.0 \end{array} \end{array}$$

where *n* is the total number of those schemas that contain the object perception and that have been executed at least twice, *T*_*s*_ is the time step when a schema *s* was last executed and *T*_*c*_ is the current time step. If schemas containing the object perception have not been executed more than twice or the object perception never appeared in the schema(s) then τ_2_ = 0 and habituation for the perceived object remains 0. Since τ_2_ is used to calculate the habituation over the period of time steps its value increases as a schema(s) containing the object perception was executed recently, as shown in Figure 2. On the contrary, τ_2_ decreases when the object perception does not occur for a period of time steps or a schema(s) containing the object perception has not been used for a long time. Thus the overall habituation is computed by

(5) $$\begin{array}{l} Habituation=1.0-e^{\left(-5\tau_{2}\right)} \end{array}$$

Similar to novelty, the coefficient at the exponential is designed to smooth the curve for the range 0–1. Habituation is expected to increase as frequent interactions with the environment lead to the same object perceptions being captured, which in turn allows the agent to select actions that promote interactions with different areas of the environment.

#### 2.1.4. Total excitation

The total excitation is calculated by combining similarity, novelty and habituation, such that

(6) $$\begin{array}{l} \phi =\omega_{1}\times Similarity\;+\;\omega_{2}\;\times \;(Novelty\;-\;Habituation) \end{array}$$

where the weights of ω_1_ and ω_2_ satisfy:

$$\begin{array}{l} \omega_{1}+\omega_{2}=1 \end{array}$$

This allows the agent to select an appropriate object to interact with, by utilizing previous experiences associated to all objects in the environment.

In particular, novelty and habituation are directly combined as they are both related to experiences associated with the currently perceived object, whereas the similarity considers all experienced perceptions of the objects which the system has previously interacted with. By varying the weights, we can simulate different artificial infants with different preferences (e.g., novel vs. favorite toy). Applying a higher weight to ω_1_ will make the agent more likely to interact with similar objects. Whereas with higher values of ω_2_, the agent will be more likely to interact with novel or less familiar objects. This can also be seen as a preference toward exploration or exploitation. The parameters ω_1_ and ω_2_ at 0.5 will allow the robot to direct its attention toward a novel object, while keeping all other parameters constant.

Alongside the object-related excitation, the agent calculates the excitation of each schema in the system, in order to select an appropriate schema to be employed. Thus, this excitation is related to the possible actions that could be performed for each object, rather than the object perception alone. If the perception(s) in the environment following an action matches the post conditions of the schema, the execution is considered to be successful. A success rate *S*_*r*_ is maintained to record the proportion of time that the expected outcome of a given schema has been achieved. This can also be considered as a reliability measure for each schema, such that

(7) $$\begin{array}{l} \lambda =S_{r}\times e^{-1.1\frac{T_{s}}{T_{c}}} \end{array}$$

where *T*_*s*_ is the last time step on which a particular schema was executed and *T*_*c*_ is the current time step. A coefficient to the exponential power is used as a smoothing factor to obtain an exponential response over the values of the ratio between schema executions and current time. Ultimately, the final excitation for each schema is calculated by considering each object that is present in the environment, so that

(8) $$\begin{array}{l} Excitation=(\omega_{3}\times \frac{\sum_{i=1}^{m}\phi_{i}}{m})+(\omega_{4}\times \lambda ) \end{array}$$

with the weights satisfying:

$$\begin{array}{l} \omega_{3}+\omega_{4}=1 \end{array}$$

where *m* is the number of all the perceived objects, ϕ_*i*_ is the excitation of the *ith* object and λ is the particular schema's excitation. Notice that due to Equation 7, a schema that is being executed repeatedly results in a lower excitation value for λ, which in turn contributes less to the final excitation. In a similar vein, schemas that are never used become more excited than their recently executed counterparts, enabling the agent to explore the environment by performing different actions. The parameters ω_3_ and ω_4_ at the value of 0.5 will allow the robot to switch its behavior, keeping all other parameters constant.

Algorithm 1 describes the process of calculating the excitation for a given environment state, referred to as world state *WS* in the system. It computes the excitation of schemas and schema chains (to be introduced next) and returns the schema or chain with the highest excitation, following the winner takes all principle. In the case of equal excitation, schema chains will be preferred to encourage the system to explore more complex behaviors.

**Algorithm 1:**
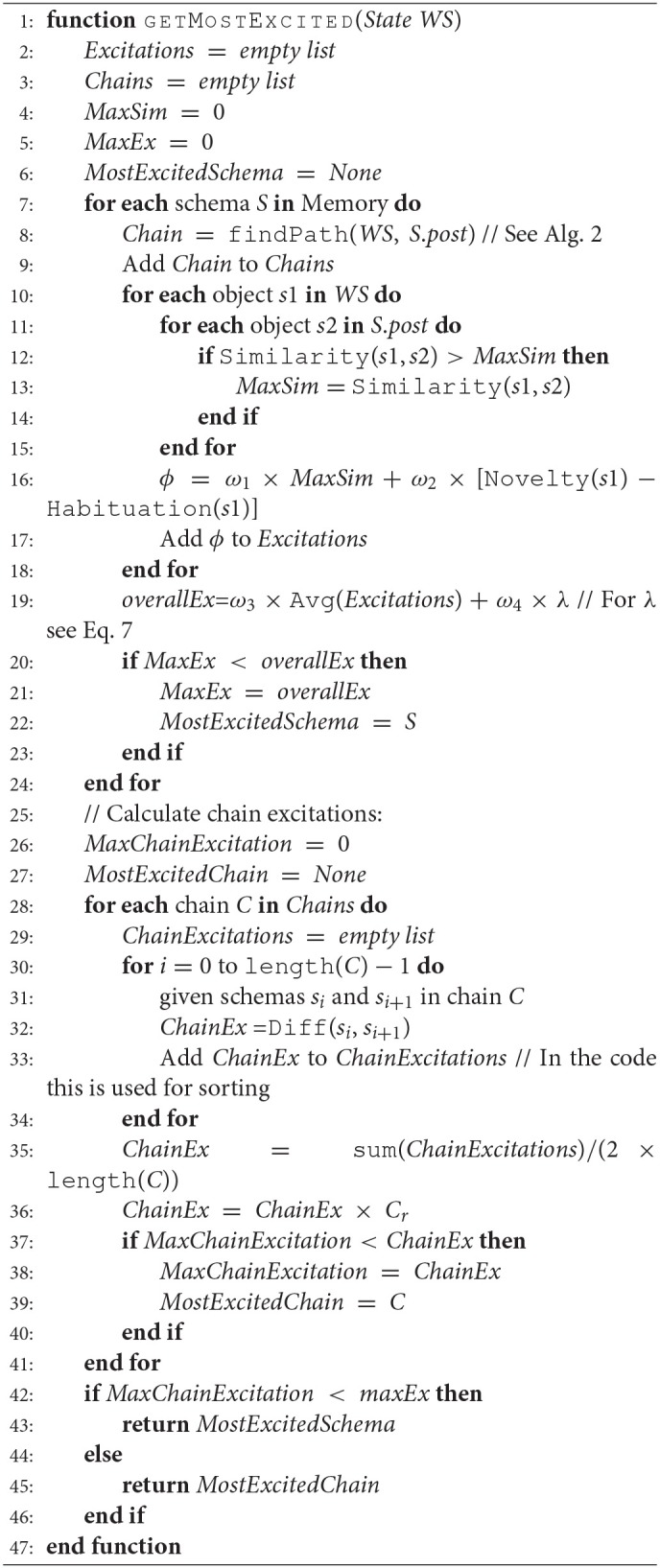
Excitation Calculation

Function Diff (line 32) returns an excitation based on the change in the preconditions of schema *s*_*i*_ to the postconditions of the next schema, *s*_*i*+1_, in the chain, and *C*_*r*_ (line 36) is the success rate of the chain. During the calculation of schema excitations, the system generates schema chains as described below in Section 2.2. Once finished, Algorithm 1 results to the schema or chain with the highest excitation.

### 2.2. Schema chains

As an agent gains more experiences and skills, certain skills can be linked together to form higher level skills in a hierarchical structure. For example, individual actions such as reach and grasp can become linked by a single reach→grasp action. Through playful exploration, more complex chains can be learned that combine basic and form more sophisticated high level actions.

**Figure 3:**
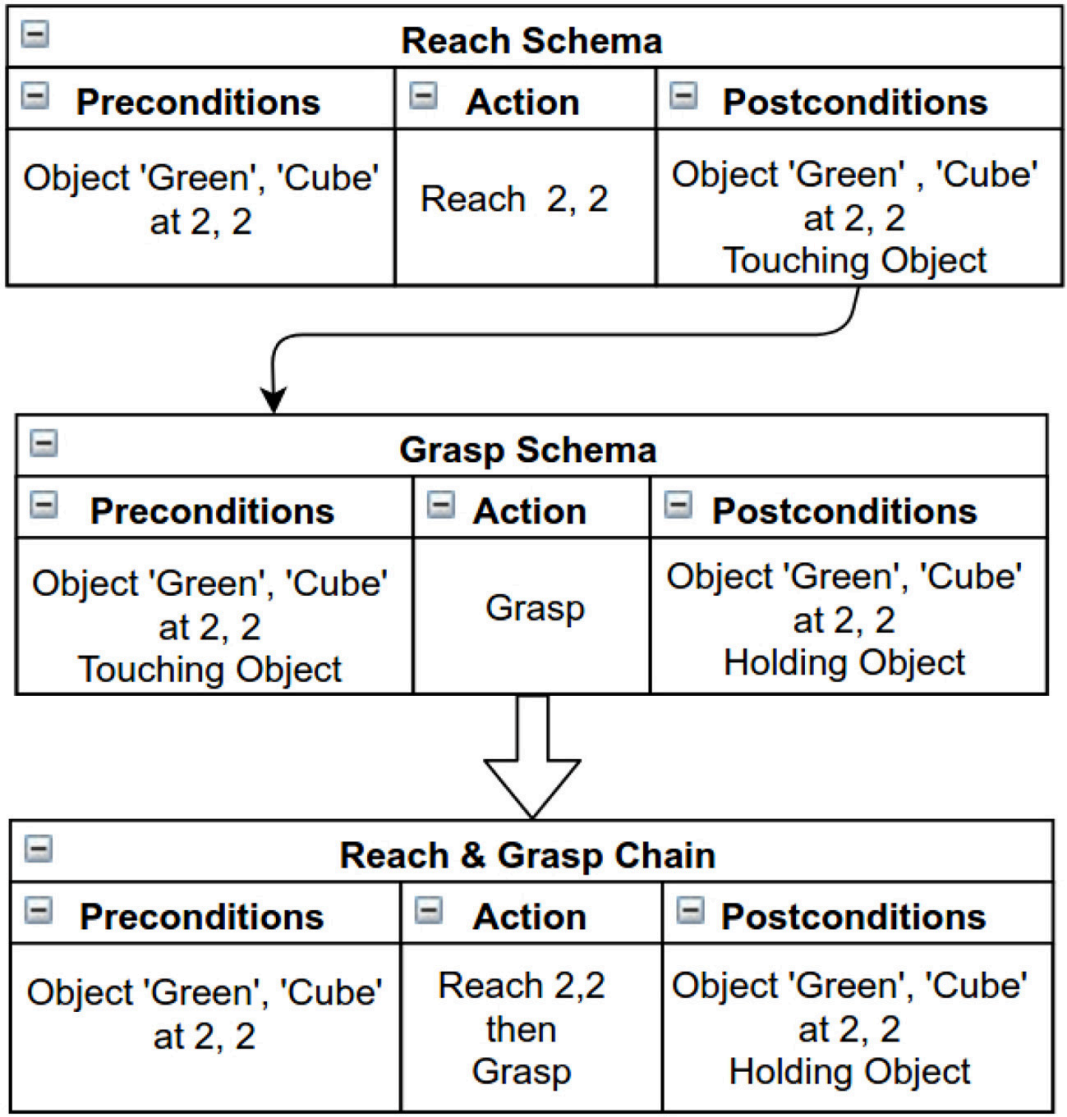
An example of chaining. Two schemas are combined to create a “2-Schema chain”.

Chains are seen as sequences of schemas, which the agent discovers by finding the links between the preconditions and postconditions of the schemas in memory. Chaining helps in achieving distant states of the environment that are not possible when employing a single schema. For example picking up an object from a reachable position needs two different actions to be achieved; (i) reach for the object and (ii) grasp it. Figure 3 shows an example of a two schema chain obtained by linking the preconditions and postcondition of two different schemas.

Algorithm 2 is responsible for the chain generation. As previously mentioned, chains are created during the process of calculating the excitation for schemas. Longer chains are discouraged during the chaining process in order to reduce computational costs and avoid overly complicated chains that are more likely to be unsuccessful. Here, a limit of 5 schemas is set.

**Algorithm 2:**
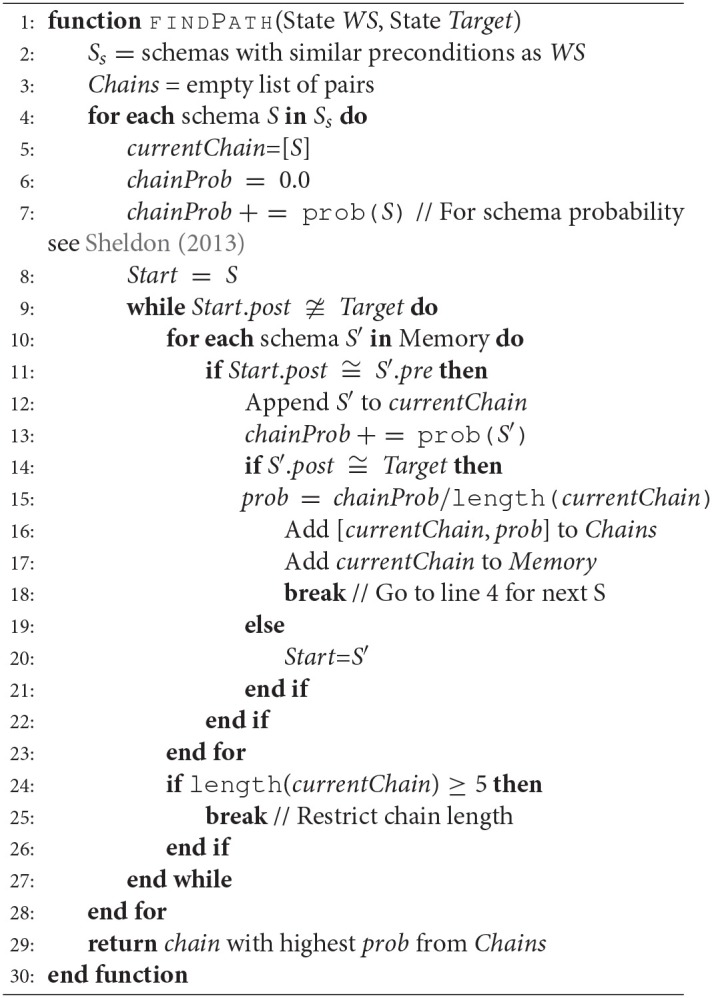
Schema chain calculation algorithm

In Algorithm 2, the schemas *S*_*s*_ contains preconditions which are a subset of the current environment, *WS*. The algorithm adds all the possible chains, for a given state of the environment, into the memory and returns the most reliable chain among them. Reliability of a chain is calculated by taking the average of success probabilities of all the schemas present in the memory.

Schemas in a chain are executed in a sequential order. A chain is considered successful if the resulting *WS* due to the preceding schema's action matches its postconditions. A chain execution is performed either as chain reflexes or motor programs as described below.

#### 2.2.1. Chain reflex

Initially chains are executed in the chain reflex mode. The world state (sensory information from the environment) is considered at the end of every executed schema in the chain. If it does not match the expected postconditions of the executed schema then the schema chain is considered unsuccessful. The term “match” means all the observations in the postconditions are obtained as an outcome. An unsuccessful chain is then opted out from the next step's schema selection.

#### 2.2.2. Motor program

If a chain is successfully executed multiple times, then it is considered reliable and therefore becomes automatic, in a sense that it behaves as a singular continuous higher-level action called a motor program. As such, the chain is used to achieve a certain condition that results from a hierarchy of actions.

At least four successful repetitions and a probability of success higher than 80% render a chain sufficiently repeatable to be considered as a motor program. Motor programs are executed sequentially without the need of intermediate verification of the world state. That is, only the last action's resulting postconditions are used for the evaluation of the motor program. Consequently, if the validation (4 successes and 80% success rate) fails, the motor program's success probability is negatively affected turning it to a standard chain.

Algorithm 3 describes the execution process of an exciting schema or a chain. Note that executing a chain is considered as taking a single time step. For further details on this mechanism of Dev-PSchema, please see Kumar et al. (2016a,b).

**Algorithm 3:**
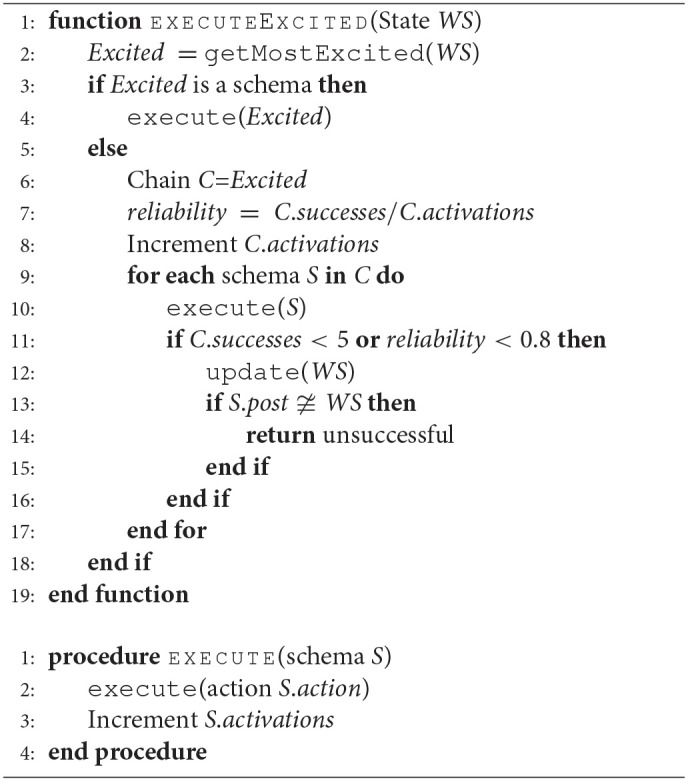
Schema/Chain execution algorithm

During the execution of a motor program, although the external state of the environment may not be directly monitored by the high-level agent, the internal proprioceptive system is active. When interfaced with a low level system that is monitoring all the sensors, the chain can still be interrupted if something unexpected was perceived.

The concept of schema chains is inspired from developmental psychology, where the ability for planning, hence action sequences, is investigated (Willatts and Rosie, 1989; McCarty et al., 1999; Rosenbaum et al., 2007). McCarty et al. (1999) investigated planning in 9, 14, and 19 month old infants. A spoon full of food was placed in various orientations in front of the infant. It was observed that 9 and 14 month old infants reached and grasped the spoon with their preferred hands. Due to difficult orientations of the spoon, 9 month old infants were found to grasp the spoon from the opposite side of the spoon, i.e., the food rather the handle side, before a corrective grasp change was required. The 14 month old infants always made corrections to make sure that the food reaches the mouth, whereas the 19 month old infants were found to switch to their non-preferred hand when the orientation of the spoon was difficult. The authors identified a series of planned strategies employed by the infants each with the goal of eating the food that can be considered as chains of action schemas.

The concept of a motor program is also inspired from developmental psychology, such as the work by Lashley (1951) investigating the hierarchical organization of behavioral plans. He believed that the concept of a motor program was being ignored over the concept of chain reflexes. The theory of chain reflexes proposes the serial order of behaviors with sensory feedback, which contributes to the excitation for each of the sequential building blocks of the chain. However, the motor program theory proposes the serial order of the actions in the behavior where the sensory feedback of the intermediate actions are ignored. Lashley (1951) believed that more time was spent at the beginning of the sequence with a shorter time in between the behavioral elements where errors in the behaviors support the theory of motor programs. The longer time spent at the beginning provides the planning of the entire sequence leading to shorter gaps between the behavioral elements, which are not sufficient to receive feedback and plan the next step. More recently, his work has been reviewed by Rosenbaum et al. (2007). Although the review suggests that going directly to motor programs and ignoring all sensory feedback is discounted, key-frames are identified in behaviors between where motor adaptation can be performed. The authors also observe that the execution time of actions between key-frames is significantly reduced following 4 to 6 repetitions. The behaviors displayed between these key-frames could be considered as short chains being executed as our motor programs, supporting the need to limit the length of any chains generated.

### 2.3. Experiments and results

We present two different experiments that demonstrate the capabilities of the proposed schema system. In the first experiment, we show the impact of varying the four weights used in the excitation calculation during play. As previously mentioned, this is equivalent to simulating different behaviors by different individuals in infancy. In the second experiment, we examine the capacity of the system with respect to playful exploration and the application of chaining, is performed both in a simulator and with a real iCub humanoid robot (Metta et al., 2008).

**Figure 4:**
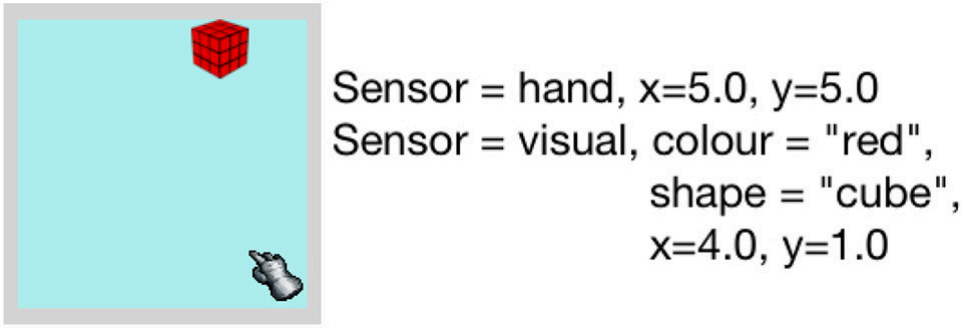
**Left:** Simulator environment containing end-effector, and object. **Right:** Description of perceived sensory information of the environment.

In the simulator, the environment consists of a 5 × 5 grid of regions, where an end effector that represents a hand and several objects are situated. Both objects and the end effector occupy one indexed region in the environment, but objects can form a stack where multiple objects occupy the same region/position. The positions of the regions in the environment are labeled with horizontal and vertical coordinates x and y respectively. An example of the simulated environment is shown in Figure 4.

Dev-PSchema receives sensory information as observations representing relevant sensor data e.g., visual observation represents color and shape. An object is represented by an observation containing relevant sensory information from a single world position in the case of the simulator and single gaze position in iCub. There are three different sensor cues simulated; visual, touch and proprioception. The visual sensor provides object perceptions that include the color, shape and the coordinates of the objects in the environment as the properties of the perception. These properties are used in similarity calculations of perceived object for excitation. The touch sensor provides indication of contact between the hand and the objects and, coupled with the proprioception is used to determine whether an object is being held. The proprioceptive sensor provides the coordinates of the hand on the grid and a value of 0 or 1 that represents the state of the hand's grip, where 0 represents the open hand and 1 represents the fully closed hand. The simulator returns all the perceptions of the objects present in the environment, alongside proprioceptive and touch perception, if touch perception exists.

The end effector can perform several different actions in the environment resulting in different perceptions. Both the actions and sensory perceptions are specified at a higher level to maintain the focus on playful interaction rather than the low level sensorimotor control. Actions used in this work are defined as follows:

- Reach x y: A reach action with parameters x and y that define the indexed position in the environment to which the hand is expected to reach. If the hand is holding an object, it is expected that the object will be moved to the x, y position by the system. If the hand is empty and moves to an x, y position where an object exists, the hand will receive a touch related perception. The touch perception varies according to the object being perceived.
- Grasp: This action initiates a grasp on an object. It is expected to close the hand and to result in grasping and holding an object at the current hand's position. If there is no object present the hand will fully close. In both cases, the corresponding touch related perceptions are expected to be captured.
- Release: This action is the reverse of a grasp. It triggers the hand to fully open and, if the hand already holds an object, to drop it on the surface of the grid. A dropped object is expected to be found at the same position of the hand, offering corresponding touch perceptions.

Although simplified, this set of actions are sufficient to demonstrate the playful capabilities of the agent similar to an infant's play. They provide an initial set of predefined actions here to bootstrap the process. In a developmental system these actions could be learnt through a combination of reflexive and exploratory behaviors.

### 2.4. Experiment 1 (A): novel vs. familiar preference

This experiment is inspired by the study of “Young childrens preference for unique owned objects” by Gelman and Davidson (2016). The study investigates the infants' preference to be attracted by a well known object (a favorite toy) rather than a new identical object or a novel, non-identical object. In the study, most of the time infants tend to select their own objects when they are given a choice of two. Interestingly, the infants are found to select the identical or novel object when they are asked to select an object for the experimenter.

To replicate the behavior of infants in the experiment only the reach schema, hence action, is used. The agent's preference is expected to be demonstrated by utilizing several reach related schemas that are gradually learned by interacting with the objects on the grid.

At first a single object, a red cube is presented to the agent. The environment and the perceived world state are shown in Figure 4. With the single reach schema in memory, the agent is most excited to interact with the object by reaching toward it. Once reaching is performed successfully, we reset the environment and return the hand to its initial position. The experiment is divided into two stages: Stage one is for familiarization, that is the agent reaches for the same object for at least three times. Stage two is for test condition, where both the familiar and a novel object are presented to the agent. This stage is further divided into four parts for each of the novel object introduced. For each object combination, the weightings for similarity, novelty and habituation, ω_1_ and ω_2_, are varied to show the change in preference. Note that ω_3_ and ω_4_ remain 0.6 and 0.4, respectively, in all the variations of this experiment. A slight weighting bias is given to the value of ω_3_ over ω_4_ to keep excitation dependence on the similarity and habituation/novelty rather than schema statistics.

### 2.5. Experiment 1 (B): action preferences

By varying the excitation parameters described in Section 2.1, several different behaviors emerge from interacting with the environment. In particular, here we vary the weights ω_3_ and ω_4_, keeping ω_1_ and ω_2_ constant (0.5 each). We examine the agents preference to either favor recently executed actions or switch to different actions during a series of executions. For this experiment, we use the same agent and the environment described in Section 2.4. However, the agent will have only two different actions here, “Press” and “Squish”, which produce the same outcome in the environment. Having the same outcome/postconditions for both actions gives the same similarity and novelty/habituation. Hence excitation of both schemas will only depend upon the schema statics.

We only use one object in the environment for this experiment to control the variation in object excitation, and place the end-effector at the same position as the object to remove the reach action from this experiment. Each action, squish or press, responds with a new observation, press, in the environment. By producing the same outcome for each action this will provide the same value for the similarity and novelty/habituation pair, so the two action will be comparable using schema excitation only. We let the agent play with the object using the actions and record which action is selected at each execution.

### 2.6. Results of experiment: 1 (A)

Following the familiarization stage, along with the original object (i.e., the red cube) we introduce four different objects one by one. Each of the new objects contains at least one common property to the red cube, such as color or shape^3^. A blue cube, a red ball, a red cube and a blue ball are used, with the latter being the object with no common properties to the object the system is familiar with. We expect that the agent will prefer to reach for the novel object when it is introduced. However, by changing the parameter values, we expect the agent will reach for the familiar object rather than the novel one. The initial weight for similarity ω_1_, and novelty and habituation ω_2_ are both set to 0.5, then the weight ω_1_ is increased in steps of 0.1, whilst maintaining ω_1_ + ω_2_=1 until the observed behavior flips toward the familiar object. Below is a discussion of the observed behavior of the agent, following the initial experience and perceiving the novel object over different values of the excitation parameter.

#### 2.6.1. Novel object with no change in the parameters (same color & shape)

**Figure 5:**
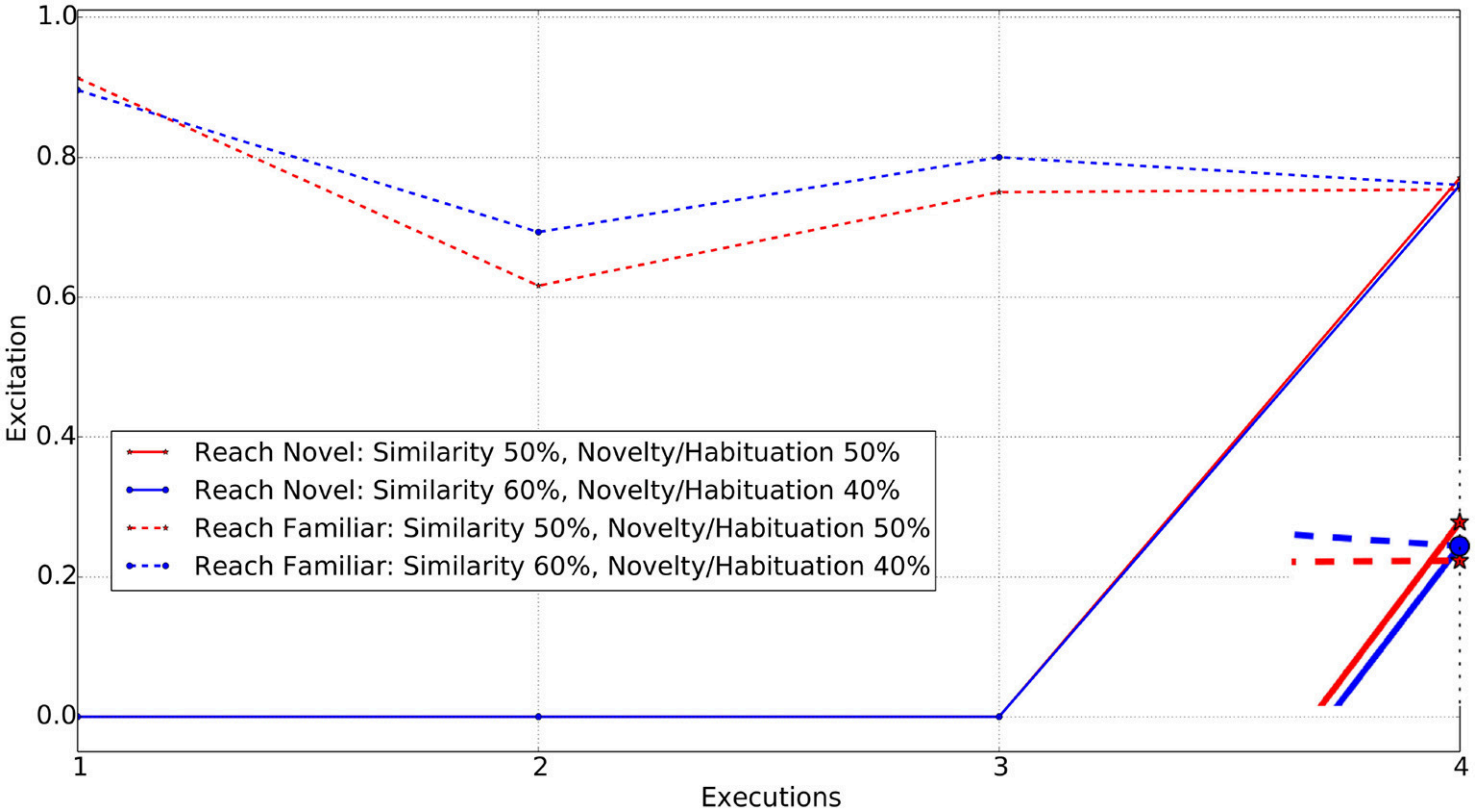
Reach actions for Familiar vs. Novel (identical in color and shape) object. Enclosed Figure shows the excitations at the 4*th* execution.

When an identical object^4^, to the red cube is placed in the environment, the agent draws its attention to it, as similarity and novelty/habituation are equally weighted. With just 10% increase in the similarity weight (ω_1_ = 0.6), the agent's preference switches to reaching toward the familiar object. Figure 5 shows the excitations of the two reaching decisions, one toward the familiar object and one toward the novel (identical) one, after having only experienced reaching to the familiar object during stage one.

For each weighting, the executed action is the one with the highest excitation. The first three executions in the figure represent the familiarization stage of the experiment. The dotted lines represent the reach for the familiar object and continuous lines represent the reach for the novel object. Note that the novel object is only introduced following the completion of the familiarization stage. The enclosed figure shows that for the novel object at equal weightings (red star) the excitation of the “reach for novel” object is higher, whereas with a similarity weighting of 0.6 (blue circle), the excitations are almost the same, giving a marginally higher value for “reach for familiar object.” At this point, the agent prefers to reach for the familiar object rather than the novel one^5^, unlike it did previously when weights were 50% for both ω_1_ and ω_2_. Thus, increase in the weight for similarity (ω_1_) enabled the agent to prefer the familiar object rather than novel.

#### 2.6.2. Novel object with change in single parameter

By varying just ω_1_ from 0.5 to 0.7, it is observed that the agent interacts with the novel object, i.e., the blue cube or the red ball after being familiarized with the red cube. Changing ω_1_ to 0.8 and ω_2_ to 0.2, the agent's behavior switches from interacting with the novel object to interacting with the familiar one after being familiarized. Here interacting, it means reaching toward the object.

**Figure 6:**
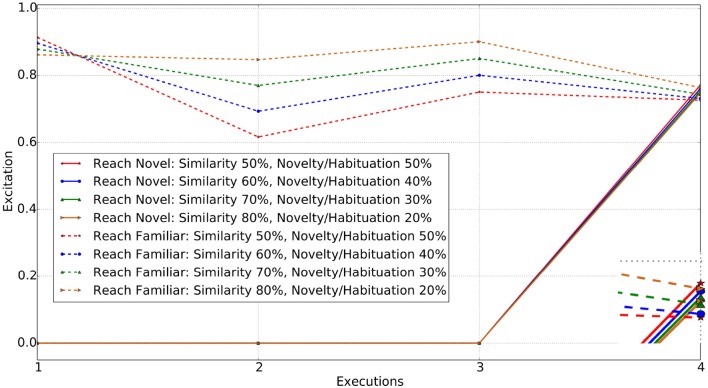
Reach actions for Familiar vs. Novel (change in either color or shape) object. Enclosed Figure shows the excitations at the 4*th* execution.

Thus the additional variation in the object properties results in the agent interacting with the novel object instead of the familiar one, until a higher weighting toward the similarity parameter is applied to draw the agent's attention toward the familiar object. At this level (similarity weight ω_1_ = 0.8), the low weight to the novelty/habituation parameters (ω_2_ = 0.2) counters the excitation generated from the different properties. Figure 6 shows the excitation of the “reach novel vs. familiar object” schemas for the different values of the excitation parameters.

Changing the similarity weight value allows several individuals to be simulated. For weights in the range 0.5−0.7 for ω_1_, the agent is found to interact with the novel object, however each of those has different excitations for reaching toward the novel object and reaching toward the familiar object actions. When the similarity weight is set to 0.8 or above, the agent is more likely to interact with the familiar object rather than the novel one. As anticipated, both the object and schema excitation weights (i.e., ω_3_ and ω_4_) cause the agent to habituate with the same object and action in case that the agent is allowed to interact with the world for a longer period of time.

#### 2.6.3. Novel object with change in both properties (color & shape)

**Figure 7:**
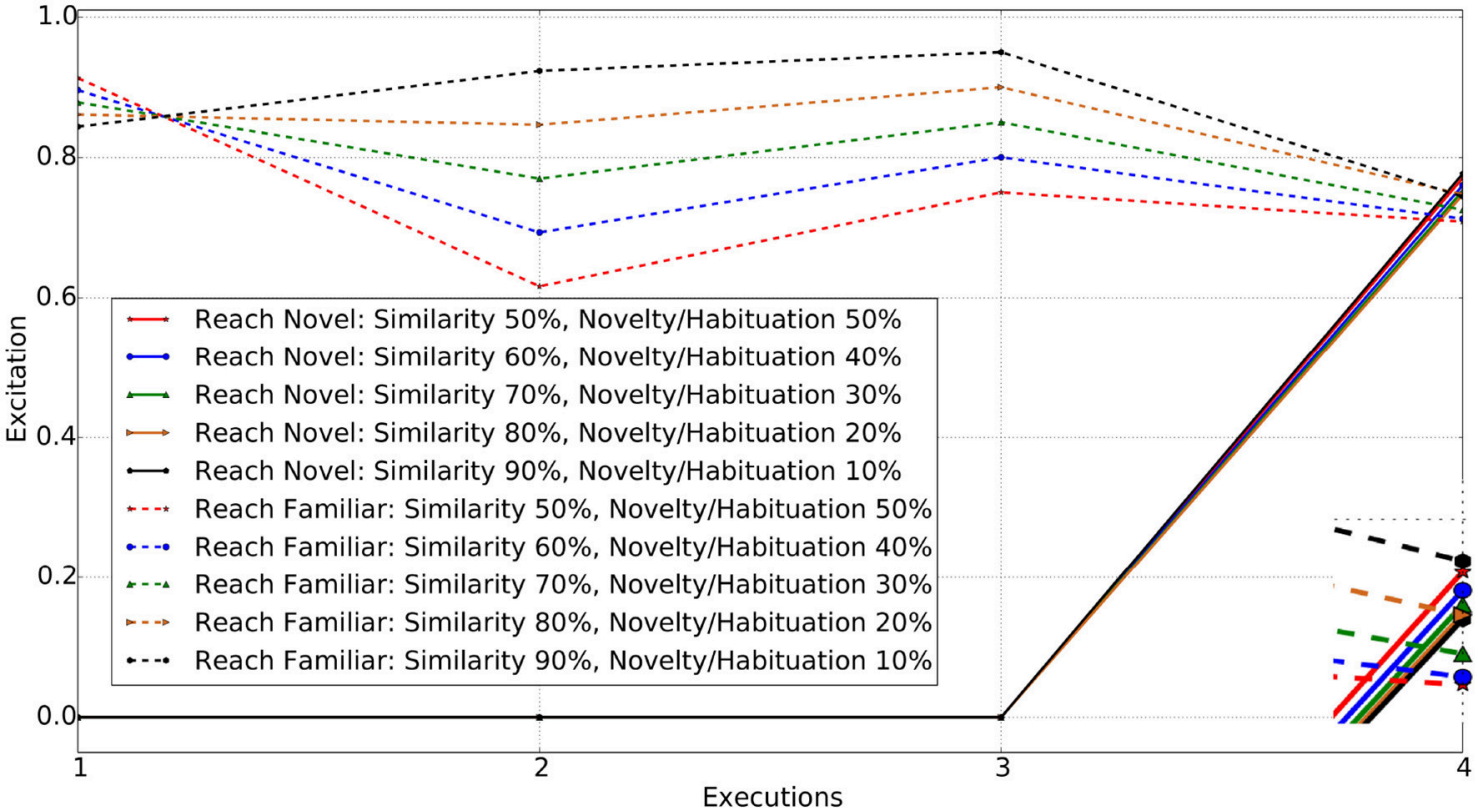
Reach actions for Familiar vs. Novel object (changed in both color and shape). Enclosed Figure shows the excitations at the 4*th* execution.

When an object with different color and shape properties to those previously experienced is introduced, the agent requires a greater weighting on similarity values in order to draw its attention to the familiar object. A completely novel object being introduced generated a high level of excitation triggering interaction with it. The simulation results show that the agent reaches for the novel object with similarity weight set to 0.5, 0.6, 0.7, and 0.8 respectively. When set to 0.9, the behavior of the agent finally switches to interest in the familiar object instead of the novel one. Figure 7 shows the excitations for schemas for reaching toward the familiar and novel objects.

From Figures 5–7, it is evident that the agent's preference in the environment changes with the variation in the excitation weights ω_1_ and ω_2_. A weighting bias toward ω_1_ will increase preference toward familiar objects. However, as the difference between the familiar and novel object increases, so do the weighting toward ω_1_.

### 2.7. Results of experiment: 1 (B)

**Figure 8:**
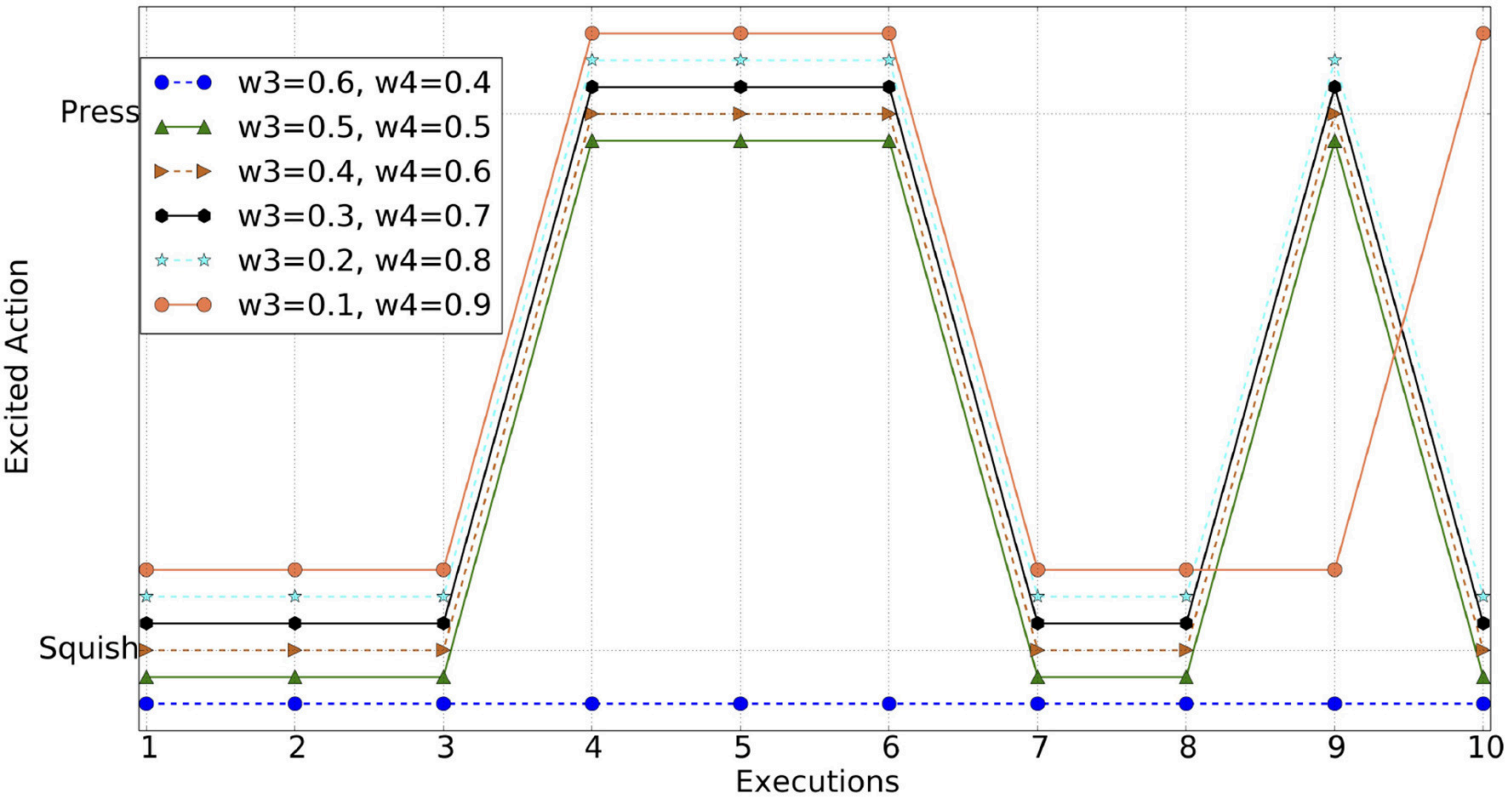
Excited schema action for different values of ω_3_ and ω_4_. Lines off-set for visibility.

In this experiment, the agent has the option to perform two different actions on the object. Both actions are controlled to provide the same outcomes in order to ensure they both provide the same object excitation based on similarity, novelty and habituation. Thus, the excited schema (or excited action) depends on the schema excitation as described in Equation 7 and its weight (ω_4_). The agent's observed behaviors for different values of the ω_3_ and ω_4_ are shown in Figure 8.

In particular, this figure shows the most excited schema, hence the action, for each execution at different paired values of ω_3_ and ω_4_, for 10 executions. From the results, it is evident that the agent shows different behaviors as the weights vary. As the weight shifts toward ω_4_, the agent becomes increasingly inclined to frequently switching between actions, rather than to explore the effects of the previous action further.

### 2.8. Experiment 2: playful discovery of action sequences-chaining

In the second experiment, we demonstrate the capability of playful behavior for exploring an environment, discovering action outcomes then creating schemas chain to form higher level behaviors in a hierarchical manner. For the first part of this experiment we will use the same environment with different objects and all the actions as described in the beginning of Section 2. The experiment is then repeated on an iCub humanoid robot (Metta et al., 2008) to show the application of Dev-PSchema in a real world scenario.

**Figure 9:**
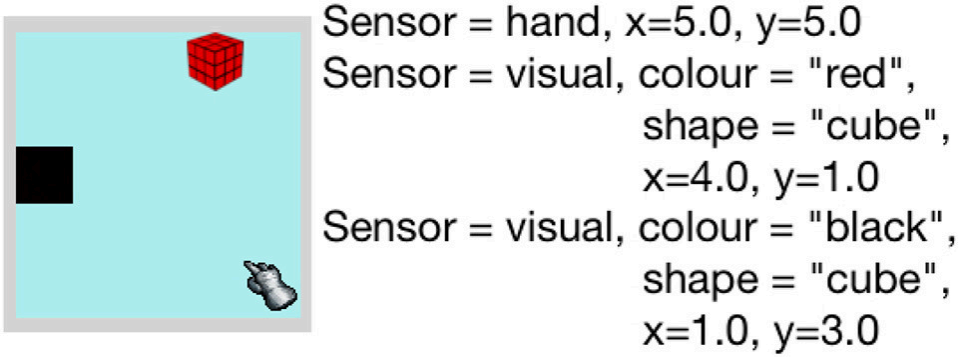
**Left**: Simulator environment containing end-effector, hole and an object. **Right**: description of the sensory information of the environment.

This experiment contains two stages. In the first stage, we introduced an object (red cube in simulator) and a hole in the environment and let the agent play with it. The hole in the environment is perceived as an object with color and shape, however it cannot be interacted with through grasping. The agent will not get any touch perception when it reaches toward it and when attempting to grasp, the hand will close fully to a fist. When an object with the similar shape as the hole is released in the hole, it disappears from the environment. Figure 9 shows the environment for this stage of the experiment and perceived state by the agent.

During the aforementioned first stage, the agent is allowed to freely play with the objects in the environment. The stage ends when the agent drops the object in the hole. Note that the aim to drop the object in the hole is decided by us (experimenter), but not specified to the agent. The agent is neither programmed with this aim, nor contains any schema to perform this specific action. At the start, the agent only contains the raw actions (Reach, Grasp, Release), without any understanding of the effects that the actions will have on either object in the environment. We expect that during a period of playful exploratory behavior using high-level motor babbling, the agent will be able to achieve the aim of the experiment i.e., creating sequences of actions.

In the second stage of the experiment, the environment is reset to evaluate the ability of the agent to exploit the knowledge gained during stage 1 and to apply chains of higher level actions. We anticipate that the agent will be able to create a chain of four actions (reach for cube, grasp, reach for hole, release) to pick and drop an object in the hole in a single execution rather than the exploratory play it did in the first stage. Note that the agent is still able to generate and reuse chains as during the first stage of the experiment. The parameter weights used in this experiment for the simulator are 0.5, 0.5, 0.6, and 0.4 for ω_1_, ω_2_, ω_3_ and ω_4_ respectively. We made a slight change in the weights of the ω_3_ and ω_4_, as compared to the weights used in Experiment 1, to encourage the agent to become habituated with schema actions quickly during play and therefore to try different schemas, hence different actions.

#### 2.8.1. Humanoid robot and environment

The above experiment was also conducted using the iCub humanoid robot (Metta et al., 2008), where a low-level system is responsible for (i) providing high-level action commands, and (ii) preparing and maintaining visual, proprioceptive and tactile perceptions. The only changes made to Dev-PSchema were to the weights and slightly increasing the tolerance of similarity to account for variations from the robot sensors. The expected sensory information is undefined, enabling the system to respond to new and previously unknown states or actions that may become available from the low level system. This shows the ability of Dev-PSchema to be applied to different and more complex settings.

In terms of actions, the reach, grasp and release commands are available after they are learnt using developmental approaches as documented in previous research efforts by Law et al. (2014b), Shaw et al. (2015), and Lewkowicz et al. (2016). Reaching is learnt by employing an approach that is inspired from hand regard in children during infancy (Rochat, 1992). This learning approach consists of random arm movements that trigger eye saccades on the visually stimulating hands. Once fixated, mappings are learned between the reaching space and the visual space, i.e., the gaze space of the robot (Giagkos et al., 2017b). Further information regarding learning the reaching space is found in Earland et al. (2014). The result of learning associations between reaching and gaze spaces is twofold. First, the robot is capable of performing reaches to a given set of coordinates within its reach space. Second, by knowing the exact hand position in the reach space, the robot is able to know where the hands are located in its gaze space by following the associations previously learned. Thus, the robot is able to perform eye and head movements in order to visually visit its hands, if necessary.

For grasping, the robot currently employs a mechanism inspired by the reflexive grasping in infancy (Giagkos et al., 2017a). When a touch sensation occurs on any tactile sensitive area of the hand, motor commands are sent to all digit joints to form a power grip. Joints that have reached a maximum are excluded from further motor activity. Digit joints are excluded when tactile sensation is constantly received from the associated fingertip, indicating that an obstacle is firmly grasped. Equivalently, a release command opens the fingers iteratively, as long as their joints have not reached their minimum values.

All perceptions are prepared by monitoring and grouping information that is received from the robot's sensory cues. At the beginning of the experiment, the robot is given time to visually explore its intermediate space by performing saccades to stimulating targets. In this experiment, green and red patches on the retina visually attract the robot's attention. Coordinates of the fixation target are calculated by considering the kinematics model of the robot with respect to the head configuration of the fixation. The gaze coordinates act as the equivalent of the world coordinates in the simulator. Subsequently, all color information that is found within the foveal area of the retina (i.e., the circular area depicted in **Figure 11**), as grouped as part of the same visual perception. This is because at this stage, visual targets that are found in the fovea are considered being part of the same object in the world. Along with the HSV color model values (i.e., Hue, Saturation and Value), the area is also calculated, being followed by the fixation target's depth. HSV is preferred over other color models such as RGB due to its robustness toward external lighting changes, with Hue varying relatively less in real-world environments. In brief, raw images from the DragonFly2 cameras of the robot are processed to identify stimulating targets of interest. Color detection is achieved by comparing the perceived HSV values against the range that defines each detectable color. Subsequently, the centroid of each target, the mean HSV and also the area's size in pixels are reported. This approach allows the system to identify potentially stimulating areas in the scene and utilize their attributes to characterize them. The low-level feature extraction mechanism employed in this experiment is discussed in Giagkos et al. (2017b). Although the gaze space is two-dimensional, an estimation of the depth of the fixation is measured to enrich the information about the visual perception in the three dimensional space. Depth is calculated after the eyes converge or diverge to perform both eye fixation.

As with the visual perceptions, tactile information is analyzed by the low-level system, in order to prepare tactile perceptions for Dev-PSchema. A tactile perception consists of the touching hand identification and the areas that received tactile information on it (i.e., the 5 fingertips and the palm). Finally, proprioception perceptions are sent for each hand of the robot, consisting of the position of the hand in the gaze space and the value related to the current hand grip. The latter reflects the hand's open and close configuration in percentage with 0% defined as fully open and 100% as fully closed.

Unlike the simulator, where the world state is provided by the software, visual changes in the real-world cannot be fully captured unless the robot visually revisits the areas of interest. Previously generated visual perceptions may no longer be available due to several real-life phenomena. For instance an object is perceived differently while it is partially or fully hidden from the eye cameras while the arms move within the reach space, or when the object has moved while an action is performed. Not all the visual perceptions are found in the retina at all times. This means that substantial head movement may be required in order to update their information, or the robot needs a way to update the world state perceptions after each action. To tackle this practical issue, the low-level system keeps a short term memory of the gaze targets with which it previously engaged, and iterates through them at the end of every action. Having such access to up-to-date world state perceptions and actions, the associated Dev-PSchema mechanisms can efficiently operate.

**Figure 10:**
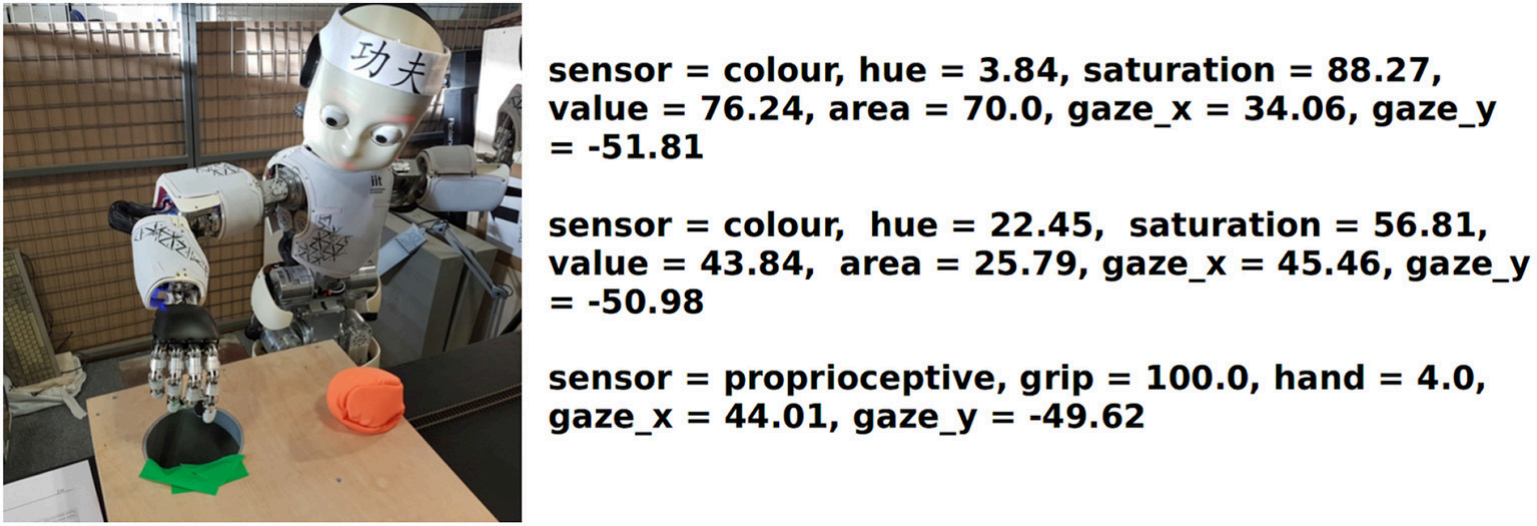
Experimental set up for the iCub and perceived sensory information.

**Figure 11:**
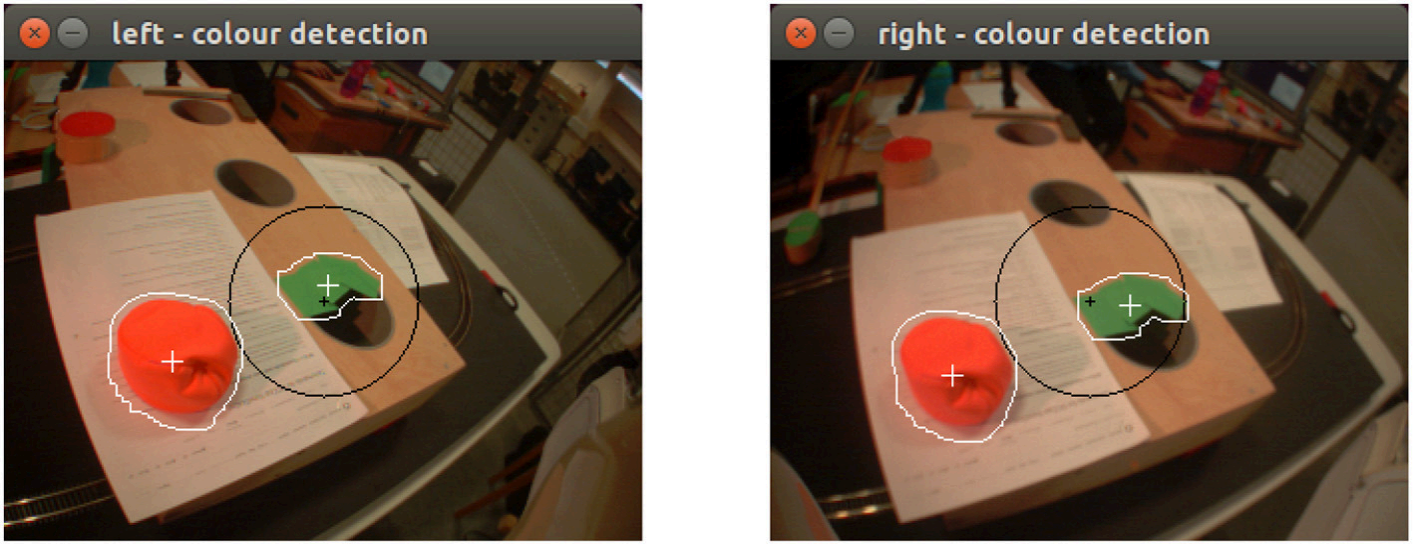
Perceived color patches by the iCub form the left and the right eye.

The experimental set-up that is used for this experiment is illustrated in Figure 10. A red soft toy is placed on a wooden board that contains a hole, big enough to ensure a successful drop. The hole is marked with a green color tape to be visible to the robot. Visual perceptions of both targets are sent to Dev-PSchema containing their coordinates in the gaze space. In order to match the simulator's experiment, one robotic arm is utilized, limiting the amount of proprioceptive and tactile perceptions to the right hand only. Figure 11 shows how targets are perceived by the eye-cameras from the environment. Using the iterative mechanism mentioned above, the visual perceptions of both the red and green targets are updated to constitute a fresh world state for Dev-PSchema's postcondition matching and excitation computations.

In this experiment we used 0.5 for all the parameter weights (ω_1_, ω_2_, ω_3_, and ω_4_). Equal weights (0.5) for the similarity and novelty/habituation pair will encourage the agent to interact with the less habituated and more novel object, having the same similarity. For ω_3_ and ω_4_, this encourages the agent to switch objects and schemas, hence actions, frequently. Values from iCub perceptions were all normalized to the range of [0, 100], with 10% tolerance to account for noise from the raw sensors.

### 2.9. Results of experiment: 2 (simulator)

**Figure 12:**
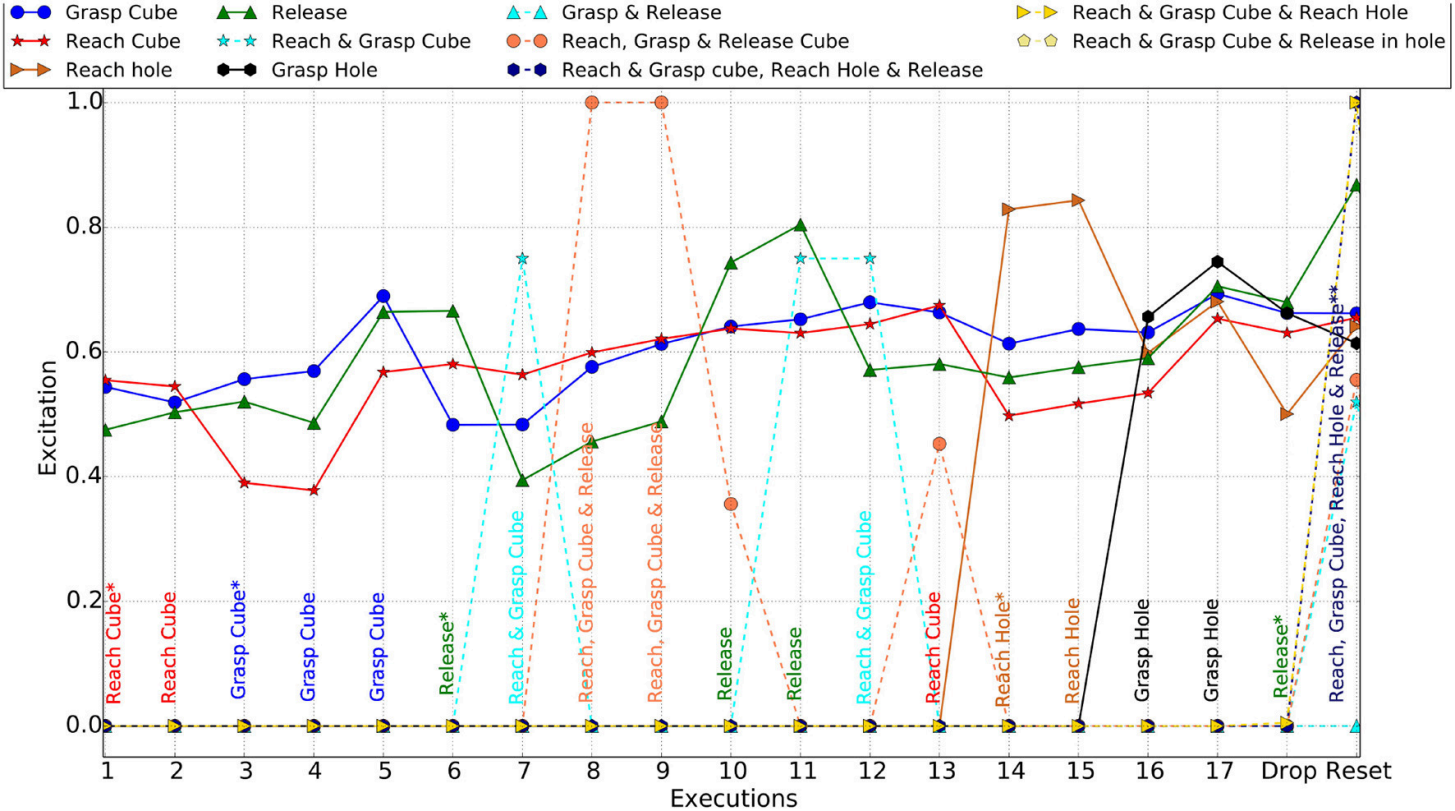
Schema and Chain excitations (Simulator). The most excited schema/chain at each execution is specified across the bottom. Actions with asterisk(*) are the positions at which new schemas emerged and with asterisk(**) are the positions where generalized schema created. The chains contain emerged schemas only. Each solid lines shows excitation of the corresponding schema, whereas dashed line shows that of a chain.

During the first stage of the experiment, the agent playfully explored the two objects and actions available in the environment. As new experiences were gained, new schemas describing these were formed. These new schemas had high novelty and therefore, where often selected as the next action, resulting in a playful behavior that repeats interesting actions, thereby also confirming their effects. Initially the agent focused its attention on the cube, learning the effects of reaching, grasping and releasing it. These actions were then combined into various chains that were tested, before the attention switched to the hole. At this point it was still holding the object, which it discovered to have moved with its hand. Attempts to grasp the hole made no difference, allowing the release action to become most excited again, and finally dropping the object in the hole. Figure 12 shows the excitations of different schemas and chains created during the playful behavior.

Before each action execution, the agent calculates the excitation of all the actions with the action (schema) of the highest excitation executed. Figure 12 shows the winning action at each execution in the experiment. During the play, it also created and executed chains of schemas. The continuous lines Figure 12 shows the excitations of the schemas and dotted lines represent chain excitations. Initially there are no chains available for the agent. Once the agent performs the grasp action, it created the “Reach and Grasp chain” and executed this at the 8*th* execution. Similarly, once the agent released the object, it discovered the “Reach, Grasp, and Release” chain. The chain was then executed twice as it had the highest excitation at the 9*th* and 10*th* execution.

Once the agent reached the first stage aim, we reset the environment, for the second stage of the experiment, by placing the object back at the same position as shown in Figure 9, and the hand back to its starting position. At this point the agent already had experience of dropping the object in the hole, so this stage evaluates the agent's ability to reuse that knowledge. The agent created the 4-schema chain “Reach, Grasp Cube, reach Hole and Release” following stage 1. It calculated the excitations of all the schemas and the chains and this 4-schema chain (dropping cube in the hole) was found to be the most excited. This is due to it being a new chain and also making the highest difference within the environment. Execution 19 (Reset on X-axis) in Figure 12 shows the excitations of all the schemas and chains for the given environment.

Figure 12 also shows that at the final execution, the excitations for all the schemas were less than the 4-schema chain. However, two other 3-schema chains i.e., “Reach, Grasp Cube & Reach Hole” and “Reach, Grasp Cube, and Release at Hole” have the same excitation as the 4-schema chain. The agent, in this condition, picks the longest chain (4-schema chain) to execute. During the chain execution, the agent checks the sensory feedback to confirm if it is getting the expected postconditions at the end of the action in the 4-actions (schemas) chain. Thus, the chain is executed in the “Chain Reflex” mode here.

### 2.10. Results of experiment: 2 (iCub)

**Figure 13:**
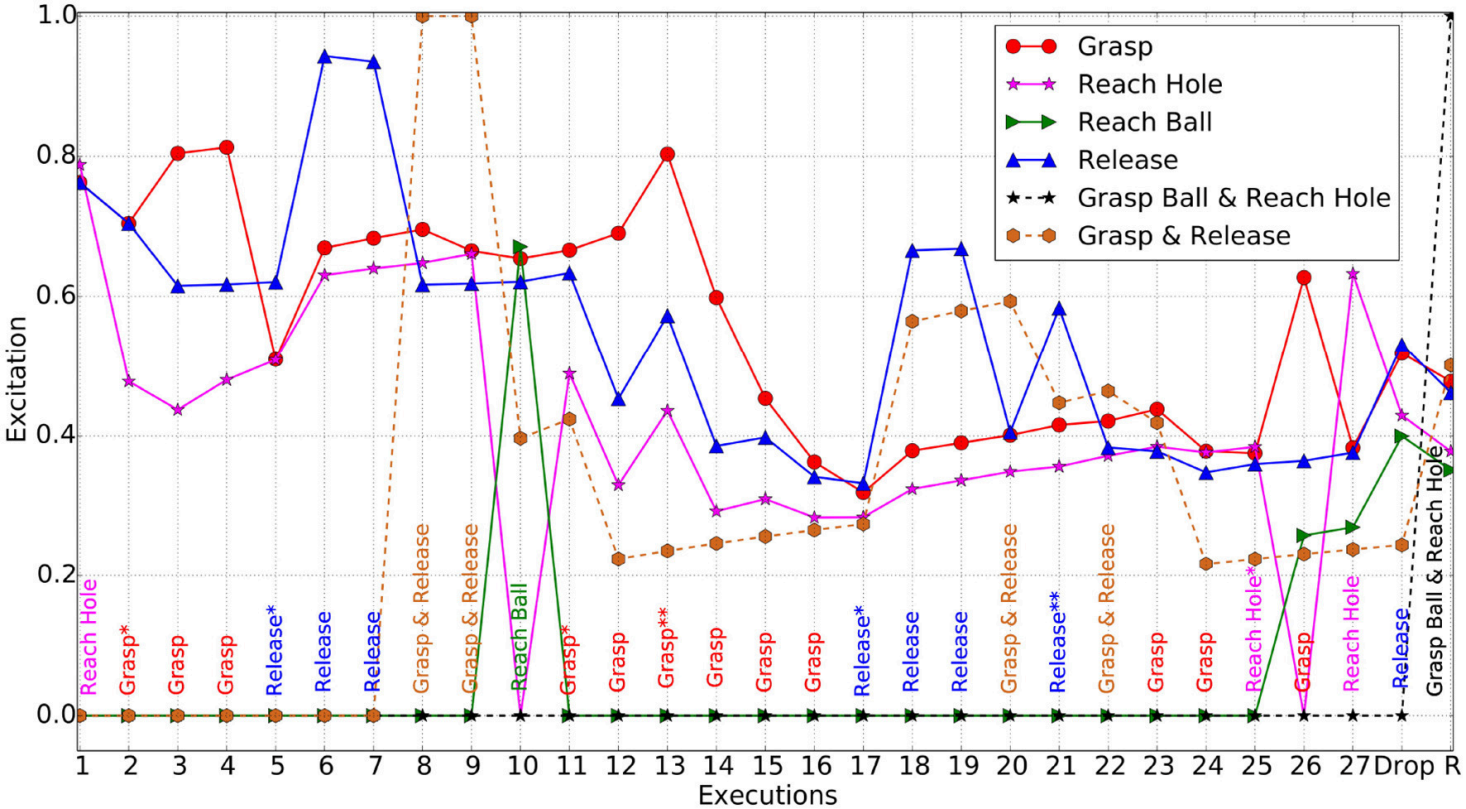
Schema and Chain excitations for the iCub. The most excited schema/chain at each execution is specified across the bottom. Actions with asterisk(*) are the positions at which new schemas emerged and with asterisk(**) are the positions where generalized schema created. The chains contain emerged schemas only. Each solid lines shows excitation of the corresponding schema, whereas dashed line shows that of a chain.

The experiment starts with the robotic arm at what we refer to as the home position. Having the arm raised next to the head and thus outside the robot's visual field, it is ensured that the initial acquired visual perceptions reflect a world state of inactivity. At the beginning, both targets are equally exciting for the robot therefore it initially selects to reach toward the hole target. Grasp happens to be the next exciting action to be performed, and due to the perception changes at both visual and proprioceptive levels, new schemas are generated. These new experiences are repeated and followed by a release action, an order which leads to the creation of a schema chain in the system; “Grasp→Release.” The related excitations are depicted against the Y-axis of Figure 13, whereas the X-axis shows the order of schema (i.e., action) execution.

After a number of executions where related to the hole target, habituation occurred and therefore the robot reached toward the ball (10*th* execution). After a successful grasp action, the world state was updated with the red ball to be ultimately perceived differently due to the grasping hand partially covering it. Subsequently, the sudden change to the visual perceptions offered a lot of new stimulation, fostering the creation of new schemas. As a result, grasping again became the most exciting action to perform, while holding the object. This repeating behavior is akin to squeezing an object, which in turn results in to several changes in visual and proprioceptive perceptions. However, after a number of grasp actions, the system habituated and a release was selected for the 17*th* execution.

Once released, the object dropped on the wooden board again giving different visual perceptions. A new post-release schema reflecting the new world state was learned for iCub to repeat, and after a few executions, it ultimately utilized the “Grasp→Release” chain to interact with the object. The robot then moved its arm to the hole coordinates while holding the ball at the 25*th* execution, followed by a release command being issued at the 28*th* execution which caused a successful drop of the ball into the hole.

For the second stage of the experiment the robot is expected to utilize the previously learned schemas and schema chains to express the similar playing behavior. Thus, without specifying a particular goal the aim is to evaluate the ability of the system to link past experiences and actions from its repertoire with the environment and to succeed in dropping the ball into the hole. The robot's performance differs in this stage from the simulator. The amount of noise in the real world is found to play an important role in delaying the process of appropriate schema selection for execution. The significant variation between schemas makes it difficult for the robot to directly link between them. However, it is anticipated that with generalization over the variation in perceptions, the generation of a full chain for dropping the ball in the hole would be possible given sufficient time for exploration. Although the number of schemas will gradually increase over time with more exploration, the process of generalization will limit the number of schemas in total (Law et al., 2014a; Kumar et al., 2016a). Thus, given the noise in the perception of the real environment, we anticipate that an affordable number of executions will be needed to achieve the desired chain. Nevertheless, subsets of the desired full chain are generated and repeated by the system, such as the “Grasp→Reach” and “Grasp→Release” chains.

## 3. Discussions

Dev-PSchema is expected to provide an interesting and appealing approach for developmental robotics. To demonstrate the abilities of the system we performed two sets of experiments.

### 3.1. Discussion of experiment 1

In the first experiment (1A), the agent is shown to express different behaviors for the novel object, while the weights of the similarity and excitation parameters change. A summary of the points at which the changes occur is given in Table 2.

**Table 2:** Summary of the weightings at which the observed behavior changed preference from familiar to novel.

**Matching properties**	**Behavior change**
	**Sim / Nov (ω_1_ / ω_2_)**
Two	0.6 / 0.4
One	0.8 / 0.2
Zero	0.9 / 0.1

Excitation and attention are seen as important factors for individual behaviors in developmental psychology. Although vision is the least developed sense at birth, humans have evolved to rely heavily on this sense (Slater and Bremner, 1989). Colombo (2001) considered alertness, object features, spatial orientation and endogenous control as the basic factors that affect visual attention in the environment. In this work, we are concerned with the last three factors of visual attention. Object features and relevant spatial orientation are inseparable. That is, the question “what” is related to the question “where” in the visual field. When the eyes fixate a target, moving it from the peripheral to the foveal vision, the direction of attention shifts in order to maintain the attention locked on the target and thus to engage with it. The endogenous control factor in visual attention is responsible for holding the attention and engaging. The novelty-familiarization pair is used in developmental psychology to investigate visual attention in humans. To investigate visual attention in this experiment, the simulated infant is initially familiarized (habituated) with a visual stimuli or event and is then presented with novel and familiar objects side by side (Wilcox, 1999; Sann and Streri, 2007; Schmuckler et al., 2007; Gelman and Davidson, 2016).

A habituation paradigm is widely used in developmental psychology experiments to test infants' ability to identify or recognize objects (Wilcox, 1999; Sann and Streri, 2007; Schmuckler et al., 2007), or events (Rosander and von Hofsten, 2004; Kellman and Spelke, 1983) based on visual cues. These examples show that infants tend to look longer toward novel objects or novel and unexpected events than toward those which are familiar or predicted. However, infants have been observed to have favorite objects for interaction and play (Furby and Wilke, 1982; Jonsson et al., 1993). Also, it has been observed that young children prefer their favorite toy over new toys, irrespective of identical form (Gelman and Davidson, 2016). In the experiments by Gelman and Davidson (2016), young children were asked to select a toy from a choice of their own or a new toy (identical and non-identical). They preferred their own toy when they were asked to choose for themselves and preferred the novel object when they were asked to select for the experimenter.

Sigman (1976) investigated the exploratory behavior of the pre-term and full-term infants at the same conceptional age. Both birth groups were familiarized with an object, a small ball. Following the familiarization period, the infants were provided with the same object along with other novel objects, each for 1 min. It was observed that both groups explored the novel objects more than the familiar objects. However, the pre-term infants explored the familiar object for longer than the full-term infants.

Ruff (1986) examined behaviors of 7-and 12-month infants with a set of objects over a period of time. Six different objects were presented in front of each infant, each for a period of 1 min. Different activities such as examining, mouthing and banging, were recorded during the trails. It was observed that the examining of each object decreased over the period of time. In addition, examining occurred before the other activities when a new object was presented. Furthermore, the 7-month old infants spent more time on examining and mouthing than the 12-month old infants.

From these examples it is evident that children show different behaviors for novel and familiar objects depending upon their experiences. This effect was reproduced within Experiment 1 by changing weights of the excitation parameters. The results showed the capability of the system to demonstrate different behaviors when interacting with a novel vs. familiar object. When the features match, the habituation effect from the first object can be considered as transferring to the new object, resulting in low novelty-excitation. Therefore, only a small change in favor of the similarity triggers a change in observed behavior. However, as the novel object becomes increasingly different, the novelty/habituation value of it becomes increasingly higher, requiring a greater weighting on similarity to cause the change in behavior.

This behavior of the artificial agent can be compared with the infants' behaviors. Steele and Pederson (1977) investigated the effect on visual fixation and manipulation with toys across 10 continuous trials in 26 weeks old infants. They were presented the same toy for the 1*st* to 7*th* and 10*th* “trails” and a novel object was introduced in the 8*th* and 9*th* “trails”. Fixation and manipulation times were found to decrease at each trial. However, fixation time was increased at the 8*th* trail when a novel object was introduced, different in either color, shape, texture or shape and texture. Similarly manipulation time was increased when the novel object contained different shape and texture. However, the manipulation time was found to continuously decrease when the novel object only differed in color.

While given of the parameters were controlled, particularly in Experiment 1b, within the pairs of weights, a higher weighting on ω_1_ will drive the agent to spend longer exploring the same object, and a higher weighting on ω_4_ will encourage the agent to try different actions. By adjusting each of the weights, different behaviors can be simulated. This could be considered as modeling different infants preferences, or different external conditions under which the agent is acting. Currently the weights are fixed at the start of an individual experiment, but in the future allowing the agent to vary these, could generate a shift from exploratory play behavior to more exploitative or focused behavior.

### 3.2. Discussion of experiment 2

In the second experiment, both agents (simulator and iCub) have shown the capability of playfully exploring their environment to discover object-action behaviors and construct hierarchical actions through the use of schema chains. In the first stage, both agents demonstrated the exploratory play behaviors in their respective environment. The second stage highlights the exploitation capability of the agents, by demonstrating direct re-use of their behaviors, learned during the exploratory play. The chains in the experiment shows the high level behaviors containing several steps in between to achieve a more distant state, i.e., postconditions of the last schema in the chain.

The simulated individuals in our experiments, have shown the capability of generating action sequences to achieve further states in the environment. The action sequences are obtained through exploratory play behavior. This behavior is followed by the exploiting behavior in stage 2, where either of the agents re-used the learnt behaviors to attain the state in the environment which was obtained previously with individual actions selected on the basing of excitation at each time step to reach the experimenters aim of dropping ball in the hole. The two modes of the chain executions, in the execution mechanism of the agents, are modeled on the “Chain reflex” and “Motor program” theories discussed as previously.

## 4. Conclusion and future work

In this work we have presented a schema-based play generator for artificial agents inspired by Piaget, termed Dev-PSchema. With experiments in both a simulated environment and with the iCub robot, we have demonstrated the ability of the system to create schemas of sensorimotor experiences from playful interaction with the environment. In particular, the proposed model captures concepts related to similarity, novelty and habituation, as a result of the agent interacting with objects, leading to the expression of different exploratory behaviors.

The first experiment has demonstrated the variations in the behaviors of the agent by changing the weights of parameters (ω_1_, ω_2_, ω_3_, and ω_4_). Experiment 1(A) illustrates the variation in behaviors of the agent by changing the weights of the similarity and novelty/habituation pair (ω_1_ and ω_2_), while keeping the object and schema excitation weights constant (ω_3_ and ω_4_). Similarly, experiment 1(B) demonstrates the variations in behaviors of the agent by changing the weights of the object and schema excitation (ω_3_ and ω_4_), keeping similarity and novelty/habituation weight pair (ω_1_ and ω_2_) constant. This aspect of the system enables us to simulate different individuals with individual behaviors rather than a single simulated agent with average behavior. It also enables the agent to switch behaviors from exploratory to more focused behavior and vice versa.

With the experimental results reported above, we have demonstrated the capability of the proposed learning system, Dev-PSchema, to simulate different individuals. By varying the weights of the excitation parameters, the agent has shown different preferences within the experimental environment. For instance, regarding the second experiment, the agent's preferences were based on its experiences in the environment, while fixing other model parameters. We have focused on the excitation mechanism and its parameters to demonstrate its importance in the agent's behaviors. The agents' behaviors show attention, interest and their preferences in the environments.

The second experiment, presented in Section 2.8, has demonstrated the play behavior of the agent in the environment and examined the potential effects of the actions on different objects. The agent was able to create a new schema while grasping the ball in the simulator, and multiple different grasp schemas were learned by the iCub due to changes in perception and the environment. For both the simulator and the iCub, the agents did not create any new schemas for grasping the hole as this does not make any change in the environment. This behavior shows that the agent is capable of learning the effects of its actions on different objects. Thus the agent learns the behaviors with objects through exploration. Furthermore, the agent reuses learnt schemas during the exploitation stage. This stage reflects the sensorimotor stage of Piaget's theory (Piaget and Cook, 1952), where infants are described as re-using or repeating their learnt behaviors involving their bodies on the interesting objects.

The second experiment also demonstrated the capability of the system to be integrated with different platforms, transferred from the simulator to the iCub robot in a laboratory environment, without making any changes to the system. In both experiments we demonstrated that the agent shows playful and exploratory behaviors. While Dev-PSchema also enables the agent to simulate different individuals with different preferences, within the current system, weights of the excitation parameters remain constant during a run and all properties are weighted equally in the object excitation.

In the future, extensions to the system will be carried out to allow the agent to dynamically adjust the weights and to learn the importance of different properties of the object, such as shape vs. color (Kumar et al., 2016a), in order to adjust the property weights accordingly. In addition, we plan to further develop the generalisaton mechanism to address the noise associated with real-world environments.

We also intend to develop the capability of the system to create and use chains with generalized schemas, so that the chains can be utilized with novel objects and in different situations. We will develop the system to exploit the chains as a high level action in different chains creating chains of chains. The chaining system will be improved further to provide an optimal action/schema sequence to achieve a user-defined target state. This will help to evaluate learning by testing the systems ability, in an effort to find solutions for user-defined problems using schemas learnt through play behaviors. In extension, we further plan to develop the system to learn from demonstrations and interactions with the other agents (human or robot). Alongside this, the system will be developed to generalize the properties of the objects and learn their limitations. For example, with the generalized reach schemas, the agent could be expected to learn the limits of the reach space in the environment.

## Author contributions

Work is predominantly based on the Ph.D. by SK supervised by PS. AG and RB are responsible for the low-level system for the iCub robot described in sections 4.6 and 4.8 as part of a project managed by PS, ML and QS. All authors can contributed to the editing of the paper.

### Conflict of interest statement

The authors declare that the research was conducted in the absence of any commercial or financial relationships that could be construed as a potential conflict of interest.

## Footnotes

^1^Either a simulator or a real robot.

^2^Where the parameters are numeric and the range is known, the euclidean distance can be used to give a similarity measure between 0.5 and 1.0

^3^Here the different types of properties are weighted equally.

^4^All the properties with the same value, except at different position.

^5^Winner takes all, therefore size of gap is not important.
